# A Review of Gene, Drug and Cell-Based Therapies for Usher Syndrome

**DOI:** 10.3389/fncel.2020.00183

**Published:** 2020-07-09

**Authors:** Lucy S. French, Carla B. Mellough, Fred K. Chen, Livia S. Carvalho

**Affiliations:** ^1^Centre for Ophthalmology and Visual Sciences (incorporating Lions Eye Institute), The University of Western Australia, Nedlands, WA, Australia; ^2^Department of Ophthalmology, Royal Perth Hospital, Perth, WA, Australia; ^3^Department of Ophthalmology, Perth Children’s Hospital, Nedlands, WA, Australia

**Keywords:** usher syndrome, gene therapy, cell therapy, ipscs, gene editing, adeno-associated virus, antisense oligonucleotides

## Abstract

Usher syndrome is a genetic disorder causing neurosensory hearing loss and blindness from retinitis pigmentosa (RP). Adaptive techniques such as braille, digital and optical magnifiers, mobility training, cochlear implants, or other assistive listening devices are indispensable for reducing disability. However, there is currently no treatment to reduce or arrest sensory cell degeneration. There are several classes of treatments for Usher syndrome being investigated. The present article reviews the progress this research has made towards delivering commercial options for patients with Usher syndrome.

## Introduction

Usher syndrome is a group of autosomal recessive disorders characterized by congenital neurosensory hearing loss, progressive night vision impairment, and constriction of the visual field due to retinitis pigmentosa (RP). Some forms of Usher syndrome may also have varying levels of vestibular dysfunction resulting in loss of balance. It is the most common form of inherited deaf-blindness (El-Amraoui and Petit, 2014) affecting an estimated 1 in 6,000 people worldwide (Kimberling et al., 2010). Usher subtypes (1, 2, and 3; Table 1) are graded according to the severity of symptoms and age of onset. Type 1 patients are born profoundly deaf and experience pre-pubertal onset of progressive vision loss caused by RP. The majority of type 1 patients also have developmental motor delays caused by vestibular dysfunction. Type 2 patients have mild to moderate congenital hearing loss with RP diagnosed during puberty. Hearing loss in type 3 patients is progressive and post-lingual, while RP onset may be delayed until mid-adulthood (Reiners et al., 2006). The clinical presentation of RP begins with night blindness caused by the degeneration of rod photoreceptor cells. Subsequent constriction of the visual field results in a “tunnel vision” effect caused by the centripetal progression of cone photoreceptor cell loss. In classical RP, the death of cones may be secondary to rod degeneration and this may ultimately lead to complete loss of vision in advanced age (Hartong et al., 2006). Many other inherited retinal diseases are associated with deafness (Table 2) such as cone-rod dystrophy and hearing loss-1 (CRDHL1, OMIM #617236), diabetes and deafness, maternally inherited (MIDD, OMIM #520000) and Leber congenital amaurosis (LCA) with early-onset deafness (LCAEOD, OMIM #617879). This review is limited to the combination of RP and deafness, the classical presentation of Usher syndrome.

**Table 1:** Genes and proteins associated with various Usher syndrome subtypes.

Type	Subtype (OMIM REF.)	Gene	Protein	% Cases*	Transcripts	Major transcript	Exons
1	1B (#276900)	*MYO7A*	MYOSIN 7A	21	14	7,483 bp; NM_000260.4	56
	1C (#276904)	*USH1C*	HARMONIN	2	11	2,232 bp; NM_005709.4	29
	1D (#601067)	*CDH23*	CADHERIN 23	6	19	11,138 bp; NM_022124.6	71
	1F (#602083)	*PCDH15*	PROTOCADHERIN 15	3	36	6,983 bp; NM_001142763.2	48
	1G (#606943)	*USH1G*	SANS	1	2	3,558 bp; NM_173477.5	4
2	2A (#276901)	*USH2A*	USHERIN	50	5	6,372 bp; NM_007123.6	72
	2C (#605472)	*ADGRV1*	ADHESION G-PROTEIN COUPLED RECEPTOR-V1	5	37	19,557 bp; NM_032119.4	91
	2D (#611383)	*WHRN*	WHIRLIN	0.4	9	3,989 bp; XM_011518485.1	21
3	3A (#276902)	*CLRN1*	CLARIN-1	2	8	2,087 bp; NM_174878.3	6
	3B (#614504)	*HARS*	HISTIDYL-TRNA SYNTHETASE	-	16	1,948 bp; NM_002109.6	13
Modifier	-	PDZD7**					

*Frequencies were calculated in a 2019 study (Jouret et al., 2019) of 684 patients with dual vision and hearing loss. A proportion of these patients did not have mutations in any of the Usher genes tested. Candidate genes are not shown. **Denotes the modifier gene *PDZD7*, which contributes to the phenotype of Usher 2A patients through interactions with USHERIN, but is not independently causal of Usher syndrome (Ebermann et al., 2010).

**Table 2:** List of non-Usher syndromes that cause hearing loss and inherited retinal disease.

Disease	Omim reference	Gene	Retinal phenotype	Systemic phenotype/s
Cone-rod dystrophy and hearing loss 1 (CRDHL1)	#617236	*CEP78*	Cone-rod dystrophy	Hearing loss
Cone-rod dystrophy and hearing loss 2 (CRDHL2)	#618358	*CEP250*	Cone-rod dystrophy	Early-onset sensorineural hearing loss
Leber congenital amaurosis with early-onset deafness (LCAEOD)	#617879	*TUBB4B*	Leber congenital amaurosis	Early-onset deafness
Polyneuropathy, hearing loss, ataxia, retinitis pigmentosa, and cataract	#612674	*ABHD12*	Retinitis pigmentosa	Hearing loss, polyneuropathy, ataxia
Diabetes and deafness, maternally inherited (MIDD)	#520000	*MTTL1*	Macular dystrophy	Adult-onset sensorineural hearing loss and diabetes, ptosis, cardiomyopathy, myopathy, renal failure, neuropsychiatric symptoms

*See www.omim.org for further information*.

### The Genetics and Biology of Usher Syndrome

Usher syndrome is caused by autosomal recessive inheritance of mutations in Usher genes known to encode proteins involved in transmembrane adhesion, scaffolding and motor transport. Ten causative genes have so far been identified. Inheritance of hypomorphic alleles with missense mutations often causes non-syndromic deafness, while nonsense and cryptic splice-site mutations result in Usher syndrome (Ahmed et al., 2008; Bademci et al., 2016). Certain types of mutations in Usher genes may also cause non-syndromic RP (Seyedahmadi et al., 2004). Digenic inheritance of mutations in separate Usher genes has also been proposed to be causative of Usher syndrome (Zheng et al., 2005; Bonnet et al., 2011), but remains controversial (Jouret et al., 2019). Additionally, the disparity in phenotypes and progression rates between monozygotic twins is suggestive of environmental influence (Liu et al., 1999). A summary of identified genes and their proteins can be seen in Table 1.

Usher proteins are localized both to the inner ear and retina. In the inner ear, Usher proteins are present in the cochlea and vestibular organs, accounting for the balance and deafness phenotypes. Usher genes instruct the differentiation of mechano-sensitive hair cells and affect hair bundle organization during development. Functional Usher proteins are also essential for the transduction of electrical signals in mature stereocilia (Grati and Kachar, 2011; Cosgrove and Zallocchi, 2014; El-Amraoui and Petit, 2014; Pepermans et al., 2014; Mathur and Yang, 2015, 2019; Han et al., 2018). In the retina, Usher proteins are found in the light-sensitive photoreceptor neurons. They have been implicated in intracellular trafficking at the connecting cilium, which links the photoreceptor’s inner and outer segments, and facilitates the movement of phototransduction proteins and lipids to the outer segment (Liu et al., 1997; Mathur and Yang, 2015). Knowledge of the function of Usher proteins in the retina is limited by a lack of effective animal models (El-Amraoui and Petit, 2014), perhaps due to their association with calyceal processes, the microvilli which protrude from the apical region of the inner segment and surround the connecting cilium of human, but not murine, photoreceptors (Sahly et al., 2012; Schietroma et al., 2017).

### Current Trials and Pre-clinical Studies for Usher Syndrome Treatment

**Figure 1:**
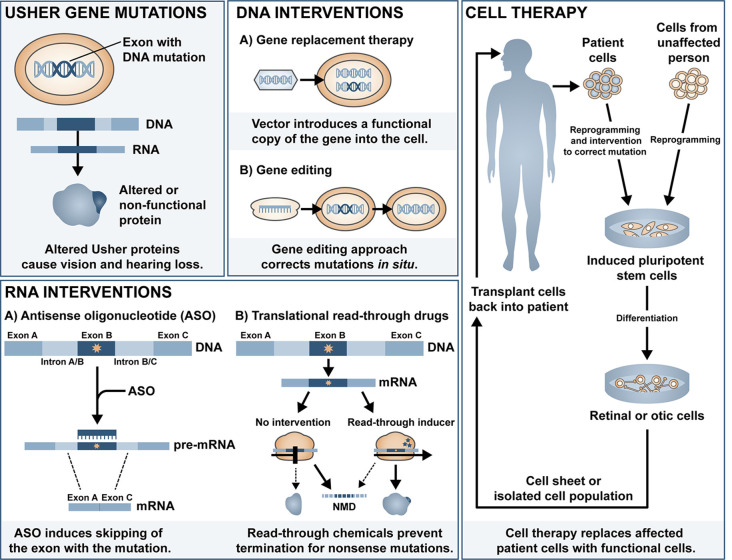
Overview of the different therapeutic approaches for Usher syndrome.

Cochlear implants are often provided to patients with type 1 Usher syndrome due to the profound deafness at birth. Those with type 2 or 3 Usher syndrome may benefit from hearing aids or cochlear implant later in life if the mild congenital deafness progresses. However, there is currently no treatment available to prevent or reverse the inevitable retinal degeneration associated with RP. There is some evidence for use of dietary supplements to delay the progression of RP, including vitamin A (Berson et al., 1993, 2018), omega-3 fish oils (Berson et al., 2004, 2012), N-acetylcysteine (Campochiaro et al., 2020)^1^ and the antioxidant taurine (Trouillet et al., 2018). Although some of these have been disputed (Rayapudi et al., 2013; Hoffman et al., 2014), the lack of strong evidence for efficacy may be related to the heterogeneity of study cohort, small sample size, and short follow-up study design. Additionally, vector-induced expression of ciliary neurotrophic factor (CNTF) was shown to be neuroprotective in a mouse model of RP (Lipinski et al., 2015), yet long-term follow up of three RP patients treated with sustained-release CNTF delivered intravitreally *via* encapsulated cell technology implant (NT-501, Neurotech Pharmaceuticals Inc.), one of which had Usher-associated RP, showed no significant difference in visual acuity (Talcott et al., 2011). In this review article, we will summarise the major classes of ongoing investigations for gene and mutation-specific treatment of hearing and visual loss and tissue regeneration and the progress and challenges in delivering therapeutic outcomes to patients with Usher syndrome. The main approaches discussed can be divided into DNA intervention, RNA intervention and cell replacement and an overview of the different approaches is shown in Figure 1.

## DNA Interventions

### Non-viral Systems

DNA interventions are possible due to engineered or recombinant vectors (broadly classified as viral and non-viral) that are synthesized to carry genetic material (Sengillo et al., 2016). Non-viral vectors (nanoparticles) are non-immunogenic, readily customizable, and may package up to 20 kb of material, allowing the delivery of large therapeutic genes (Moore et al., 2018).

Lipid-based nanoparticles, which form a protective bilayer around transported genetic components, have been explored as potential treatments for hearing loss. Zou et al. (2017) investigated the functional, inflammatory, and apoptotic response to liposome nanoparticle delivery to the murine inner ear and found no adverse reactions. Additionally, Gao et al. (2018) reported that cationic lipid-mediated delivery of Cas9:guideRNA complexes to the Beethoven (*Bth*) mouse model of deafness selectively disrupted the dominant mutant *Tmc1* allele, reducing hearing loss. Inner hair cells (IHCs) and outer hair cells (OHCs) had significantly improved survival rates and auditory brainstem response (ABR) thresholds compared to uninjected controls (Gao et al., 2018). Lipid-based nanoparticles have also been used to deliver base editing machinery in a proof-of-concept study to ameliorate hearing loss (discussed further in section Base Editing; Yeh et al., 2018).

Supraparticles are colloidal nanoparticle aggregates with a larger drug loading capacity relative to individual nanoparticles (Sperling and Gradzielski, 2017). Supraparticles have already been used to deliver the developmental neurotrophin, brain-derived neurotrophic factor (BDNF), to the inner ear of a hearing loss guinea pig model (Wang et al., 2014). BDNF is required for the maintenance of spiral ganglion neurons (SGNs; Ylikoski et al., 1993) and may serve to protect or regenerate SGNs as well as promote synaptic regeneration at the ribbon synapse (Suzuki et al., 2016). Supraparticles offer sustained longer-term release of BDNF and maintained near-wild-type numbers of SGNs in the guinea pig cochlea (Wang et al., 2014). However, delivery of supraparticles may mediate the unintentional movement of small nanoparticles from the inner ear to the cerebral spinal fuid *via* the cochlear aqueduct (Zhang et al., 2013).

Another significant advancement in the non-viral delivery platform was the development of the polyethylene glycol-substituted 30-mer lysine peptide (CK30-PEG) nanoparticles, which have been used successfully in a cystic fibrosis clinical trial (Konstan et al., 2004). CK30-PEG nanoparticles may also be effective for the treatment of ocular diseases, as they show a retinal targeting efficiency comparable to viral vectors up to 2 weeks post-injection (Farjo et al., 2006; Han et al., 2012a). Appreciable transgene expression mediated by CK30-PEG nanoparticle gene delivery has been reported in retinal degeneration models, including an autosomal dominant model of RP (Cai et al., 2010; Han et al., 2012b). In the autosomal RP model study, nanoparticles containing the mouse opsin promoter and the *Prph2* gene were subretinally delivered to *Rds* mice carrying a haploinsufficiency mutation, resulting in wild-type-level recovery of cone function but a modest restoration of rod function (Cai et al., 2010). Modified CK30-PEG nanoparticles with a photoreceptor-specific promoter also led to the structural and functional rescue of Stargardt-associated pathology in the *Abca4*^−/−^ mouse model of vision loss (Han et al., 2012b). Recent studies have used solid lipid nanoparticles (SLNs) as a more efficient non-viral delivery system and a study on a mouse model of x-linked juvenile retinoschisis showed transduction of retinal pigment epithelium (RPE) and photoreceptors and improved retinal phenotype (Apaolaza et al., 2016).

Though nanoparticle delivery is promising and continues to be developed, there are several roadblocks to its clinical application, including biodegradability, biocompatibility, and non-specific transfection (Yin et al., 2014; Chen et al., 2016). Topical delivery is the simplest and most patient-friendly form of administration. However, ocular barriers and the long diffusion pathway prevent therapeutic levels from reaching the retina (Bisht et al., 2018). The intraocular injection route would, therefore, be preferable, though the transient expression associated with non-viral gene delivery (Bisht et al., 2018; Huang and Chau, 2019) would necessitate repeated injections and consequently, an increase in their associated risks. Thus far, no pre-clinical studies have investigated nanoparticle delivery of Usher genes but the large packaging capacity of non-viral vectors does increase the potential of this type of platform for treatment development for Usher-causing mutations in the large genes such as *CDH23, PCDH15, ADGRV1, MYO7A*, and *USH2A* (Table 1).

### Viral-Based Gene Replacement Therapy

With the advantage of increased efficiency over non-viral vectors (Nayerossadat et al., 2012), viral vectors currently represent the most promising approach to therapeutic genetic interventions. Recombinant viral vectors utilize the inherent capability of viruses for cellular transduction to deliver genetic material to donor cells *in vivo*. However, viral vectors come with their limitations, including limited packaging capacities. Potential immunogenicity is also an obstacle to clinical application, though advances in molecular biology have allowed for the separation of wild-type viral coding genes and cis-acting sequences. Consequently, segregation can now produce viral vectors that do not reconstitute by recombination into productive viral particles but still maintain viral infectivity capacity (Kay et al., 2001).

Gene replacement therapy involves the introduction of a non-mutant copy of the affected gene to restore expression to the host cell or tissue (Sengillo et al., 2016). One of the first described, and most frequently used vectors, adenovirus, has a large (~40 kb) packaging capacity and has been used in over 400 clinical trials (Wold and Toth, 2013). In a study by Bennett et al. (1996), an adenovirus-based vector was used to deliver the Phosphodiesterase β subunit gene to *Rd1* mice, delaying photoreceptor degeneration. However, many people have circulating antibodies against adenoviruses, so their use in gene therapies is limited due to a high immunogenic response (DiCarlo et al., 2018). Furthermore, adenovirus has shown to have low tropism for photoreceptor cells *in vivo* (Li et al., 1994), an additional limitation that instigated the search for other types of viral vectors with improved targeting in the eye. The sections below will discuss the two most promising vectors: lentivirus and adeno-associated virus (AAV).

#### Lentiviral Vectors

Lentiviral vectors, derived from the human immunodeficiency virus, were also initially favored by researchers for clinical application due to their relatively large packaging capacity (reviewed in Zheng et al., 2018). However, whilst their immunogenicity is low, their tendency to integrate into the genome calls into question their clinical applicability (Zheng et al., 2018). Pioneering work by Hashimoto et al. (2007) led to the development of a lentiviral-mediated *MYO7A* gene delivery vector that produced wild-type protein levels in RPE cells in culture, as well as rescuing phenotypic RPE defects *in vitro*. *In vivo*, melanosome mislocalization and opsin accumulation at the photoreceptor connecting cilium was corrected in the *Myo7a*-deficient *shaker1* mouse model of Usher 1B (Hashimoto et al., 2007). Subsequently, UshStat, a therapeutic recombinant vector expressing the human *MYO7A*, was developed using the equine infectious anaemia virus (Zallocchi et al., 2014), a non-pathogenic, non-primate-derived lentivirus capable of transducing human cells (Mitrophanous et al., 1999; Mazarakis et al., 2001). Subretinal delivery of UshStat to *shaker1* mice was shown to protect photoreceptors from light-induced degeneration and demonstrated tolerability in non-human primates, which led to this approach progressing to a clinical trial for the prevention of RP in Usher 1B patients (NCT02065011; results pending).

#### Adeno-Associated Viral Vectors (AAV)

Safety is of principal concern in all viral vector technologies and as AAVs are not known to cause any human pathogenesis, they have emerged as the vector of choice for gene therapy applications worldwide. AAVs have low immunogenicity, high cellular specificity, and long-lasting gene expression. AAVs typically persist as episomes [Zheng et al., 2018; circular genomes which replicate independently of their host (Watanabe and Fukasawa, 1961)], reducing the risk of insertional mutagenesis. Vandendriessche et al. (2007) directly compared lentiviral and AAV vectors and found that AAV serotypes 8 and 9 induced higher expression of transgenic factor IX protein than lentiviruses in mouse models of hemophilia B and severe combined immunodeficiency, without interacting with proinflammatory cytokines. AAVs also hold potential for the treatment of Usher retinal degeneration as they can efficiently transduce photoreceptors and RPE (Rodrigues et al., 2018). Successful treatment of vision loss in LCA type 2 (LCA2) and choroideremia *via* AAV-mediated delivery of the *RPE65* and *CHM* genes, respectively, have been demonstrated in human clinical trials (reviewed in Russell et al., 2017). The LCA2 trials have now resulted in the first-ever Food and Drug Administration (FDA)-approved and European Medicines Agency (EMA)-approved AAV-based gene therapy drug for LCA2 due to recessive *RPE65* mutations (Luxturna^®^ or Luxturna™/voretigene neparvovec-ryzl or voretigene neparvovec, Roche and Novartis).

A major disadvantage of AAV vectors is their relatively small (4.7 kb) packaging capacity (Zheng et al., 2018); however, *in vivo* delivery of the smaller Usher genes has been investigated using AAV vectors. Delivery of rAAV2/8 containing *USH1G* cDNA to the inner ear improved hearing and hair cell disorganization in the Usher 1G mice (Emptoz et al., 2017). Durable inner ear expression of Usher genes has also resulted in *in vivo* protein restoration of WHIRLIN (Zou et al., 2011) and CLARIN-1 in several studies (Dinculescu et al., 2016; Geng et al., 2017; Dulon et al., 2018; György et al., 2019).

Transduction efficiency is a crucial factor in the success of gene therapy and is highly cell and serotype-dependent. Several synthetically-produced AAV variants have been investigated for Usher syndrome treatments. One example is the synthetic rAAV2/Anc80L65, which may transduce close to 100% of IHCs and 90% of OHCs in mice (Landegger et al., 2017). Using rAAV2/Anc80L65, Pan et al. (2017) demonstrated gene and protein recovery of Harmonin in an Usher 1C mouse model. Deverman et al. (2016) reported a highly efficient synthetic vector, AAV9-PHP.B, which showed robust transduction efficiency in the retina of a dominant RP mouse model (Giannelli et al., 2017). Further, AAV9-PHP.B carrying a green fluorescent protein (GFP) reporter transduced the inner ear and retina of wild-type mice at a rate of 60–80% for IHCs, 30–40% for OHCs and 70–80% for photoreceptors (György et al., 2019). When used to package the *Clrn-1* gene, AAV9-PHP.B mediated rescue of low-frequency hearing (4–8 kHz) in a mouse model of Usher 3A deafness (György et al., 2019). However, the tropism and potential toxicity of the AAV9-PHP.B vector in the central nervous system (CNS) of non-human primates is still under study (Hordeaux et al., 2018; reviewed in Deverman et al., 2018). The potential, therefore, for treatment of Usher syndrome using certain AAV-based vectors is high, but the large size of some Usher genes, including some of the most common forms of Usher such as USH1B (*MYO7A*), does present unique challenges for the field.

#### Oversized Adeno-Associated Viral Vectors

As mentioned above, the packaging limitation of AAVs precludes delivery of several of the larger Usher genes, including *MYO7A* (USH1B; Jaijo et al., 2006) and *PCDH15* (USH1F; Alagramam et al., 2001). Some studies, however, have pushed the limits of AAV packaging capacity by using oversized AAV transgene constructs (fAAV). A study by Allocca et al. (2008) identified rAAV2/5 as being an efficient packager of up to 8.9 kb of genetic material, allowing, in theory, the large retinal disease genes *MYO7A*, *ABCA4* and *CEP290* to be packaged and delivered subretinally. They showed that fAAV2/5 *ABCA4* delivery led to a stable improvement of morphological abnormalities and retinal dysfunction associated with a mouse model of Stargardt disease. Additionally, Colella et al. (2013) identified defects in light/dark adaptation in *shaker1* mice, which was improved by fAAV2/5 delivery of *MYO7A*. However, other studies have shown that these vectors do not contain full-length genes, but instead tend to contain heterogeneous mixtures of gene fragments, most of which are truncated and typically less than 5 kb (Dong et al., 2010). Furthermore, Grieger and Samulski (2005) demonstrated that AAV vectors carrying genomes larger than 5 kb had less efficient transduction capacity due to a post entry preferential degradation of AAVs carrying larger genomes. Though the truncated genomes may reassemble before transcription and form full-length proteins inside the cell (Lopes et al., 2013), the lack of characterization and heterogeneity of this approach limits its clinical use.

#### Multi-Adeno-Associated Viral Vector Systems

As an alternative to the oversized AAV approach, different research groups have tested the use of dual and triple AAV vector systems. Multi or dual AAV systems use the inherent concatemerization of AAV genomes *in vivo* to form full-length cDNA which can then be transcribed into functional mRNA within the transduced cell (Trapani et al., 2014). Different methods are used to deliver dual/multivectors and are usually divided into trans-splicing, overlap, and hybrid approaches. Trans-splicing vectors, first described by Yan et al. (2000), separates the gene of interest into 5^′^ (left) and 3^′^ (right) halves, with the 3^′^ end of the left construct containing a splice donor site, which concatemerises with the splice acceptor site located on the 5^′^ end of the right construct. In the overlap approach, the 3^′^ end of the right half and the 5^′^ end of the left half contain a highly recombinogenic overlapping region which mediates homologous recombination between gene segments (Duan et al., 2001). This sequence can be from an external gene, like alkaline phosphatase, or directly from the gene of interest. Finally, hybrid vectors contain both the splice donor/acceptor sites of trans-splicing vectors and the recombinogenic properties of overlapping vectors, allowing reconstitution to occur *via* either method (Ghosh et al., 2008).

Several research groups have published proof-of-concept studies using multi or dual AAV systems, including for inherited retinal diseases (Colella et al., 2014; Dyka et al., 2014, 2019; McClements et al., 2019). Multi-AAV systems have been used to express *MYO7A* *in vivo* and *in vitro* with equal or higher efficiency than fAAV delivery (Dyka et al., 2014), and to produce full-length mRNA with 100% fidelity to the target cDNA (Dyka et al., 2014). Additionally, Maddalena et al. (2018) expanded the transfer capacity further by using a triple therapeutic vector system to incorporate large genes such as *ALMS1* and the Usher 1D gene, *CDH23*. Truncated protein products were detected in eyes treated with *CDH23* but not *ALMS1* triple AAV vectors. A functional response was not recorded in *CDH23*-treated mice but treated *Alms1*^−/−^ mice showed a non-significant increase in outer nuclear layer thickness and transient (2–6 months) improvement of electroretinogram a- and b-wave measurements (Maddalena et al., 2018).

Despite the promising potential of dual AAV approaches, most studies show very low levels of protein expression using this system. Dyka et al. (2014) quantified the relative expression of full-length cDNA mediated by dual AAV reconstitution and found hybridizing vectors to be 2–3 fold more efficient than overlap and trans-splicing counterparts in *MYO7A*-transfected HEK293T cells. Data published by Colella et al. (2014) found that hybrid and trans-splice dual AAV reconstitution achieved approximately one-quarter of the photoreceptor expression levels induced by single AAV vectors in the large white pig retina. In their 2018 article, Maddalena et al. (2018) reported that the co-transduction rate for triple AAV vectors was <6% of single vector expression in HEK293T cells. Interestingly, this efficiency increased when vectors were delivered subretinally to mice and pigs (photoreceptor expression = 27 ± 6% and 39 ± 17% of that induced by single AAV, respectively). In a recent study, Carvalho et al. (2017) tested the *in vitro* and *in vivo* expression of the different dual AAV approaches and found hybrid vectors to have superior rates of reconstitution. They showed that reconstitution efficiency in HEK293T cells for trans-splicing, hybrid, and overlapping vectors was 10.3%, 15.3%, and 17.4%, respectively. The efficiency of overlapping vectors was found to be gene-specific as it was dependent on the length of the recombinogenic region and showed no detectable levels *in vivo*. *In vivo* subretinal delivery to the mouse retina resulted in full-length protein expression in 9.07% and 1.78% of cells transduced with hybrid and trans-splicing vectors, respectively (Carvalho et al., 2017). Interestingly, they were the first to show a discrepancy in reconstituted mRNA and protein levels after dual AAV delivery which may be explained by transcript instability of longer mRNA sequences (Feng and Niu, 2007).

An alternative to dual AAV systems may be dual-intein splicing, which is based on protein rather than mRNA reconstitution, but is still delivered by AAV vectors. Inteins are segments of proteins that have been described as “protein introns,” as they can excise themselves from the sequence of peptides and join the flanking portions (the exteins) together. Recently, Tornabene et al. (2019) demonstrated reporter protein levels in C57BL/6J mice retinae induced by dual-intein splicing to be comparable to single AAV transduction and significantly higher than dual AAV transduction. This approach was also shown to alleviate retinal degeneration in animal models of Leber Congenital Amaurosis (*Rd16* mice) and Stargardt disease (*Abca4^−/−^* mice). Physiological symptoms of retinal degeneration, including RPE lipofuscin accumulation in *Abca4^−/−^* mice, and outer nuclear layer thinning in *Rd16* mice, were significantly reduced in the dual-intein treated mice. Additionally, pupillary light responses increased by ~20% in treated *Rd16* mice compared to untreated *Rd16* controls (Tornabene et al., 2019). However, the viability of this approach for the delivery of large genes has not yet been tested in models of Usher syndrome.

### DNA Editing

While gene delivery aims to replace a defective gene with a functional copy, DNA editing attempts to directly correct the mutation *in vivo*, which also allows the repaired gene to be expressed under endogenous regulators. Furthermore, the size of the gene is not a limiting factor. Repair of single base transitions or transversions at the DNA level can be achieved through the induction of a DNA break to facilitate incorporation of the correct DNA base (Kim et al., 1996; Christian et al., 2010; Jinek et al., 2012; Cong et al., 2013; Mali et al., 2013) while targeted conversion of a single DNA base can reverse a transitional mutation (Komor et al., 2016; Gaudelli et al., 2017).

#### Gene Editing

The first attempt using gene editing for Usher syndrome was a study by Overlack et al. (2012) that used zinc finger nucleases (ZFNs) to target the p.R31X mutation in the human *Ush1c* gene. Their *in vitro* results show partial repair of the *Ush1c* gene and recovery of harmonin protein in a p.R31X cell line after transfection with ZFNs. In recent years, however, the discovery of the clustered, regularly interspaced, palindromic repeats (CRISPR) and CRISPR-associated protein 9 (Cas9) system have changed the field of gene editing profoundly. Their higher efficiency, simplicity, and targeting capacity have made them the preferred technology of choice for gene editing (Khan, 2019).

The CRISPR/Cas9 system uses an adapted microbial immune technique for precise editing of the genome (Jinek et al., 2012; Cong et al., 2013; Mali et al., 2013). A guide RNA is engineered to target a specific locus, which is then digested by the Cas9 endonuclease (Jinek et al., 2012; Cong et al., 2013; Mali et al., 2013). Either of two endogenous mechanisms repairs double-stranded breaks in the genome. The clinically-favorable homology-directed repair relies on an intact template strand whilst error-prone non-homologous end-joining is independent of a template sequence, and often results in insertions or deletions (Adli, 2018).

CRISPR/Cas9 has been used to study potential treatments for inherited retinal diseases. Moreno et al. (2018) used the viral delivery of CRISPR components to downregulate *Nrl* in mouse models of non-Usher RP. *Nrl* is a transcriptional regulator that indirectly determines whether photoreceptors develop into rods or cones (Cheng et al., 2004; Moreno et al., 2018). The knockdown of *Nrl* expression transformed rods into cone-like cells that did not experience rod-specific degeneration. This leads to a decrease in rods and therefore night blindness, but an increase in daylight vision (Moreno et al., 2018). However, mouse models of Usher 1C and 1G have cones that are sensitive to oxidative stress, indicating that increasing the number of cone-like cells may not be a viable option for Usher RP treatment (Trouillet et al., 2018).

Viral delivery of CRISPR/Cas9 to fibroblasts derived from an Usher 2A patient showed some rescue of Usherin expression, however, the efficiency of restoration was not significant enough to proceed to clinical trials (Fuster-García et al., 2017). Additionally, induced pluripotent stem cells (iPSCs) derived from a patient with a mutation in *MYO7A* were investigated by Tang et al. (2016). Stereocilia-like protrusions from patient iPSCs developed disorganized morphology which was rescued by CRISPR/Cas9 editing (Tang et al., 2016), indicating a potential treatment avenue for Usher 1B patients that deserves further investigation.

Recently, the CRISPR/Cas9-based tool EDIT-101 received FDA approval for a clinical trial in LCA10 patients (NCT03872479), leading to the first-ever direct human administration of CRISPR/Cas9^2^. EDIT-101 is a gene editing therapeutic which utilizes a Staphylococcus aureus Cas9 guide RNA which has high specificity for the c.2991 + 1655A >G transversion in intron 26 (IVS26) of the CEP290 gene, limiting off-target effects (Maeder et al., 2019). Recently, the company behind EDIT-101, Editas^3^, has publically announced the utilization of a new therapeutic, EDIT-102, to target Usher type 2A^4^. Another recent update is the introduction of a new gene editing tool, “prime editing,” which utilizes a synthetic fusion of an altered Cas9 endonuclease and reverse transcriptase, directed by a gene editing guide RNA (Anzalone et al., 2019). This technology shows promising specificity and broad applicability to a large number of human pathogenic mutations, although further studies will be needed to confirm its viability for treating Usher syndrome.

Though the potential of CRISPR/Cas9 to make precise and patient-specific gene corrections with high targeting capacity is attractive, the efficacy of *in vivo* delivery is still in doubt due to potential off-target effects (Fu et al., 2013; Kuscu et al., 2014). Advancements in the field since its original application in gene editing, including the development of anti-CRISPR proteins to limit non-specific activity (Pawluk et al., 2016; Rauch et al., 2017; Nakamura et al., 2019), have potential to address some limitations of this technology and further studies could help validate CRISPR/Cas9-based approaches for the treatment of Usher syndrome.

#### Base Editing

The advent of CRISPR/Cas9 gene editing systems has also allowed for the development of base editing, which utilizes inactivated Cas nucleases and single-stranded editing enzymes to replace one nucleobase with another without creating double-stranded breaks (Rees and Liu, 2018). Two classes of base editing technology have so far been reported; cytosine base editors (CBEs; Komor et al., 2016) which convert C-G base pairs to T-A, and adenine base editors (ABEs), which convert A-T to G-C (Gaudelli et al., 2017). Both CBEs and ABEs introduce transition mutations between chemically similar base pairs, which can theoretically correct over 60% of human pathogenic point mutations (Rees and Liu, 2018). However, transversion base editing (e.g., G-C to C-G) remains an elusive target.

Base editing has already been used in proof-of-concept studies in mouse models of Duchenne muscular dystrophy (Ryu et al., 2018), phenylketonuria (Villiger et al., 2018), hereditary tyrosinemia type I (Song et al., 2020) and hypercholesterolemia through targeting the *PCSK9* gene (Chadwick et al., 2017). Importantly, base editing has also been used to investigate enhanced cellular reprogramming of supporting cells to cochlea hair cells to mediate hearing loss (Yeh et al., 2018). C-T conversion of a single base in the *CTNNB1* gene prevented phosphorylation and degradation of β-catenin protein, leading to a 7-fold increase of β-catenin levels in HEK293T cultures. Consequently, there was an increase in signaling to the Wnt pathway, which is crucial to the development of sensory hair cells. When delivered to mice *via* intracochlear injection, base editing resulted in the differentiation of cochlea supporting cells to MYO7A-expressing hair cells (Yeh et al., 2018).

## RNA Intervention

RNA splicing can be altered to either restore exons lost due to aberrant splicing induced by the mutation, or induce exon skipping to remove nonsense or missense mutations in coding regions. RNA intervention resulting in altered splicing is achieved through the binding of an antisense oligonucleotide to RNA strands (reviewed by Havens et al., 2013). Base editing of RNA is also possible (Bass, 2002). Finally, translational readthrough techniques aim to suppress protein truncation by overriding mutations that cause premature termination of the translation machinery. The reader is referred to a review outlining the therapeutic progress of translational readthrough-inducing compounds in the treatment of inherited diseases (Nagel-Wolfrum et al., 2016).

### RNA-Based Drug Interventions

RNA molecules designed to silence or interfere with toxic gain-of-function mutations have been investigated in the treatment of several non-Usher models of genetic hearing loss. Notably, Maeda et al. (2005) identified a short interfering RNA (siRNA) that targets the R75W allele variant in GJB2, a common cause of autosomal recessive hearing loss. The synthetic siRNA suppressed GJB2 in HEK293T cells by >80% for more than 120 h. When administered to the GJB2 mouse model, siRNA treatment suppressed GJB2 expression by >70% and prevented hearing loss (Maeda et al., 2005, 2007). Another study by Shibata et al. (2016) developed a microRNA targeting non-syndromic deafness caused by gain-of-function mutations in *TMC1*. The microRNA was packaged *via* AAV2/9 and delivered to *Bth* mice, resulting in significant preservation of hearing for up to 21 weeks relative to untreated controls. The animals who responded best to treatment maintained ABR thresholds 40 dB greater than untreated counterparts (Shibata et al., 2016).

#### Antisense Oligonucleotides

ASOs are short nucleic acid sequences that modulate gene expression *via* complementary binding to mRNA. ASOs are often synthesized to activate ribonuclease H (RNAse-H, which degrades mRNA) or target splicing defects (Goyal and Narayanaswami, 2018). ASOs can then be designed to target a pathogenic mutation; hence, the size of the gene is not an obstacle as with gene delivery techniques. However, the half-life of the drug means re-occurring invasive administration to the eye rather than one-off treatments, as is the case with a gene therapy approach.

ASO treatment has been widely investigated for retinal disease therapies (Huang et al., 2017). Recently, up to nine antisense-oligonucleotide variants were identified for the treatment of Stargardt disease caused by the intronic c.4539 + 2001G >A mutation in the large *ABCA4* gene (Garanto et al., 2019). In a separate study, screening of ASOs led to the discovery of QR-110 (Dulla et al., 2018), a therapeutic for LCA type 10 (LCA10), caused by the c.2991 + 1655A >G mutation, in the *CEP290* gene (Den Hollander et al., 2006). Like Usher syndrome, the pathogenesis of LCA10 is caused by defects in the photoreceptor connecting cilium. QR-110 was able to restore wild-type *CEP290* transcript and protein expression in mutant fibroblasts and decrease ciliopathy in 3-dimensional (3D) retinal organoids (explained further in section Cell Therapy for Retinal Degeneration). The drug was also well-tolerated in mice, rabbits, and non-human primates (Dulla et al., 2018), leading to the approval of phase I/IIa clinical trial (NCT03140969) by ProQR Therapeutics^5^.

ASOs have also been developed for autosomal dominant RP (Naessens et al., 2019), as well as Usher 2A-associated RP (Slijkerman et al., 2016). An engineered ASO targeting a pseudo-exon-causing mutation in *USH2A* (c.7595–2144A >G) displayed splice-correcting properties in patient-derived fibroblasts (Slijkerman et al., 2016). The same company that funded this study, ProQR Therapeutics, has also investigated an ASO, QR-421a, for the treatment of Usher 2A-associated RP caused by a common exon 13 mutation (c.2299delG; Van Diepen et al., 2019). The efficacy of this treatment was initially demonstrated in patient-derived retinal organoids and animal models (Table 3), with exon-skipping capability maintained in cynomolgus monkeys for more than 100 days post-treatment. They also showed that Usherin protein was present in wild-type zebrafish larvae at the photoreceptor connecting cilium but absent in untreated c.2299delG zebrafish. Partial restoration of correctly localized usherin expression was observed in treated larvae as well as improved ERG recordings compared to untreated zebrafish (Van Diepen et al., 2019; conference abstract). The results of this study have allowed for advancement to a clinical trial (NCT03780257), which has already led to the first treatment to the eyes of an Usher 2A patient using QR-421a^6^ (preliminary findings reported on the 20th of April 2020 here: https://ir.proqr.com/news-releases/news-release-details/proqr-announces-positive-findings-interim-analysis-phase-12).

**Table 3:** Summary of transgenic animal disease models discussed in this review.

Human disease (equivalent)	Model	Animal	Gene	Mutation	Therapy tested	Reference
Usher 1B	*Shaker1* (Sh1^−/–^)	Mouse	Myo7a	G720X induced by ENU	AAV2/2-mediated gene delivery	Colella et al. (2013)
Usher 1C caused by c.216G >A mutation	*Ush1c c.216G >A*	Mouse	Ush1C	c.216G >A	AAV2/Anc80L65-mediated gene delivery	Pan et al. (2017)
					ASO-mediated suppression of exon 3 cryptic splice site (ASO-29)	Lentz et al. (2013), Vijayakumar et al. (2017), Donaldson et al. (2018) and Ponnath et al. (2018)
Usher 1G	*Ush1g*^−/–^	Mouse	Ush1g	*Ush1g*^fl/fl^ mice targeting exon 2, crossed with *PGK-cre* mice	rAAV2/8-mediated gene delivery	Emptoz et al. (2017)
Usher 2A	*Ush2a*^mcm1^	Zebrafish	Ush2a	Homozygous premature stop mutations in exon 13	ASO-mediated exon 13 skipping (QR-421a)	Van Diepen et al. (2019)
Usher 2D caused by compound heterozygous Q103 ×/c.837 + 1G >A mutation	*Whirlin*^−/–^	Mouse	Whrn	Targeted deletion of exon 1	AAV2/8-mediated gene delivery	Zou et al. (2011)
Usher 3A	*Clrn1* KO	Mouse	Clrn1	Targeted deletion of *Clrn1* upstream promoter, first exon, 269 bp of the first intron	AAV-mediated gene delivery	Dinculescu et al. (2016), Geng et al. (2017) and György et al. (2019)
	*Clrn1*^ex4−/−^−/–^^	Mouse	Clrn1	Early ubiquitous deletion of Clrn1 exon 4	AAV-mediated gene delivery	Dulon et al. (2018)
	*Clrn1*^ex4fl/fl^Myo15-Cre^+/−^	Mouse	Clrn1	Hair cell-specific postnatal deletion of Clrn1 exon 4	AAV-mediated gene delivery	Dulon et al. (2018)
Non-syndromic hearing loss (DFNA36)	*Beethoven (Bth)*	Mouse	Tmc1	c.1235T>A	Lipid-mediated delivery of cas9:gRNA complex	Gao et al. (2018)
Non-syndromic hearing loss (SLC26A4)	*Slc26a4-null*	Mouse	Slc26a4	Mutations not reported	iPSC otic progenitors	Chen et al. (2018)
Non-syndromic hearing loss (GJB2)	*GJB2*_R75W-eGFP	Mouse	Gjb2	R75W	siRNA	Maeda et al. (2005)

*Abbreviations: *ENU*, N-ethyl-N-nitrosourea*; AAV*, adeno-associated virus;* ASO*, Antisense oligonucleotides; *iPSC*, induced pluripotent stem cells; *siRNA*, small interfering RNA*.

Several studies have investigated ASO-29 treatment of the Usher 1C, c.216G >A mouse model, which exhibits hearing loss as well as disrupted exploratory movements, indicating vestibular dysfunction. ASO-29 administration to the inner ear of c.216G >A mouse pups has been shown to improve both the auditory and vestibular response (Lentz et al., 2013; Vijayakumar et al., 2017; Donaldson et al., 2018). However, mounting evidence has demonstrated an age threshold for effective delivery, with early-treated mice responding better to treatment. Ponnath et al. (2018) demonstrated that both outer and inner hair cell function was effectively restored by ASO-29 treatment, but the threshold for effective outer hair cell treatment (post-natal day 5) was lower than that of inner hair cell treatment (post-natal day 7). Considering these promising results in the auditory and vestibular response of USH1C mice, it would be interesting to assess the efficacy of ASO-29 towards the vision loss phenotype. However, the limited visual phenotype of the USH1C mice model means alternative models will need to be used for testing ASO-29.

#### Translational Readthrough Inducing Drugs

Nonsense mutations cause premature termination codons (PTCs), leading to the production of a truncated polypeptide or targeting of the transcript by nonsense-mediated decay (NMD). When a translation termination codon (UAA, UGA, UAG) enters the ribosomal A site, it is recognized by eukaryotic release factor 1 (eRF1), which changes conformation when bound to eRF3, initiating a signal cascade that results in hydrolytic cleavage of the polypeptide by eRF1. Translation termination is a competition between recognition by eRF1 leading to termination and continuation of the translation process, leading to readthrough or natural suppression. Natural suppression occurs >0.1% of the time in non-mutant transcripts, but PTCs increase the rate of natural suppression to up to 1% (Keeling et al., 2012). Nonsense suppression therapies aim to increase translational readthrough, avoiding the production of a truncated protein. The two main obstacles with this type of therapy are avoiding mRNA degradation by NMD, and avoiding the promotion of non-specific readthrough (Frischmeyer and Dietz, 1999; Keeling and Bedwell, 2011; Wang and Gregory-Evans, 2015; Dabrowski et al., 2018; Campofelice et al., 2019).

Nonsense suppression has clear advantages over gene-based therapies; they are not gene- or mutation-specific, the size of the gene is not an issue, and they allow the cell to maintain expression regulation. However, they are associated with nephrotoxic (Mingeot-Leclercq and Tulkens, 1999) and ototoxic (Selimoglu, 2007) effects and often result in the incorporation of a non-cognate amino acid, which may alter protein function. A recent advancement in the field was the development of anticodon engineered transfer RNAs (ACE-tRNAs), which recognize and suppress stop codons while encoding the cognate amino acid of the non-mutant polypeptide (Lueck et al., 2019). This method induced a specific readthrough of *CFTR* mutations *in vitro*. Low levels of off-target suppression were detected, depending on the genetic environment of the mutation, but the authors suggest that endogenous cellular pathways to degrade incorrectly transcribed proteins would be sufficient to offset these effects (Lueck et al., 2019).

Several studies have investigated nonsense-mediated therapies for the treatment of Usher syndrome. Initial studies focussed on nb30, a synthetic derivative developed from the commercial aminoglycoside, paromomycin (Nudelman et al., 2006). Nb30 was shown to induce significant nonsense suppression of a common *USH1C* mutation (p.R31X) with reduced toxicity and increased biocompatibility compared to commercial aminoglycosides (Goldmann et al., 2010). Similarly, favorable toxicity profiles were observed in nb30-mediated suppression of *PCDH15* nonsense mutations relative to traditional antibiotics (Rebibo-Sabbah et al., 2007). Subsequently, a second paromomycin derivative, nb54, was identified as a promising drug candidate for nonsense suppression which demonstrated readthrough ability in several prominent disease genes, including Usher 1F syndrome (Nudelman et al., 2009). In this study, Nudelman et al. (2009) show that nb54 is capable of inducing *in vitro* stop codon suppression 3–5-fold times more efficient for *PCDH15* (Usher 1F) mutations p.R3X and p.R245X compared to three other aminoglycoside compounds.

PTC-124 (Ataluren) is another translational readthrough inducer which has been studied for application in cystic fibrosis and Duchenne muscular dystrophy cases (Welch et al., 2007), though success has thus far been limited in clinical trials (Wilschanski et al., 2011; Kerem et al., 2014; McDonald et al., 2017). PTC-124-induced readthrough *in vitro* has been demonstrated in the common p.Trp3955* mutation, which accounts for 13% of *USH2A* mutations (Neuhaus et al., 2017). Additionally, PTC-124 treatment induced *in vitro* and *in vivo* readthrough of *USH1C* mutations, leading to the recovery of protein function with superior biocompatibility to gentamicin and nb30 (Goldmann et al., 2011). Finally, Goldmann et al. (2012) compared nb30, nb54, and PTC-124 for translational readthrough of *USH1C* mutations and identified both PTC-124 and nb54 as ideal drug candidates. Though promising, functional rescue of Usher phenotypes *in vivo* will nonetheless be necessary to evaluate the efficacy of both PTC-124 and nb54.

## Cell Replacement Therapy

Cellular therapies are therapeutic approaches that aim to regenerate damaged tissue by introducing replacement donor cells (Zakrzewski et al., 2019). This approach relies on the survival of connecting interneurons when the stimulus-receiving neuron has already degenerated in the cochlea or retina. Typically, progenitor cells derived from stem cells are used as an unlimited source of donor cells. Stem cells are pluripotent progenitors that can differentiate into cell lineages from each of the three germ layers. Early stem cell studies relied on the controversial use of human embryonic stem cells (hESCs; Omole and Fakoya, 2018) until the discovery in 2007 by Shinya Yamanaka and colleagues that adult human somatic cells, such as adult fibroblasts, could be reprogrammed back into a pluripotent state (Takahashi et al., 2007). They achieved this through the expression of characteristic pluripotent markers *SOX2*, *OCT3/4*, *KLF4*, and *c-MYC*, delivered to the cell *via* retroviral vectors (Takahashi et al., 2007). As the stem cells carry the same genetic information as the somatic cells from which they were made, this breakthrough allowed human diseases to be replicated and studied non-invasively in the laboratory for the first time (Omole and Fakoya, 2018).

### Cell Therapy for Retinal Degeneration

iPSCs can aggregate to form “embryoid bodies” (aggregates of cells thought to mimic the early embryo), which can be prompted to differentiate into specific lineages (Omole and Fakoya, 2018), including 3D retinal organoids (Nakano et al., 2012; Phillips et al., 2012; Zhong et al., 2014; Mellough et al., 2015, 2019). These organoids closely resemble *in vivo* human eye development, forming eye field-like domains that form 3D retinal neuroepithelium and RPE. The neural retina and RPE domains transition into a pseudo optic cup-like structure and retinal progenitor cells differentiate into neurons including horizontal cells, amacrine cells, and retinal ganglion cells (Zhong et al., 2014). Organoids develop a defined outer nuclear layer containing radially-aligned photoreceptors with detectable inner and outer segments connected by a connecting cilium (Zhong et al., 2014; Mellough et al., 2015, 2019; Lowe et al., 2016; Parfitt et al., 2016). These photoreceptors express phototransduction proteins, including opsins, and can respond to a light stimulus (Zhong et al., 2014; Hallam et al., 2018; Mellough et al., 2019).

Retinal organoids are a good resource of photoreceptor progenitors for transplantation. These progenitors can be enriched before transplantation for a homogenous cell population *via* cell sorting for dissociated cell transplants (Lakowski et al., 2015, 2018). MacLaren et al. (2006) transplanted developing mouse photoreceptors into the retinae of degenerative mouse models with successful integration and differentiation, noting an optimal therapeutic window corresponding to the specification of rod cell fate. Subsequently, photoreceptor transplantation was reported by several research groups (Pearson et al., 2012; Barber et al., 2013; Gonzalez-Cordero et al., 2013; Singh et al., 2013; Santos-Ferreira et al., 2016), including Barber et al. (2013) who looked into six mouse models of inherited retinal degenerations (IRDs) with environmental, disease course, and genetic factors influencing the integration outcome (Barber et al., 2013). Though these initial photoreceptor transplantation experiments seemed promising, the transfer of cytoplasmic material between labeled donors and host photoreceptors has been attributed to the seemingly high rates of integration (Santos-Ferreira et al., 2016; Singh et al., 2016). Alternatively, a stem cell-derived retinal sheet may be transplanted into the recipient’s eye (Assawachananont et al., 2014). Though clinical trials have focussed on RPE transfer in patients with age-related macular degeneration (AMD) and Stargardt Disease (NCT03102138, NCT02941991, NCT01469832), none thus far have reported results on photoreceptor transplants. However, a first-in-human phase I/II dose-escalation study is currently examining the safety and tolerability of human retinal progenitor cells in patients with RP (NCT02464436, estimated completion date: July 2021). Furthermore, proof-of-principle studies in mouse and non-human primates showed survival of retinal sheets [containing hESC-derived retinal cells (Shirai et al., 2016), hiPSC-derived RPE cells (Mandai et al., 2017) or hiPSC-derived retinal progenitors (Tu et al., 2019)] post-transplantation, and improvement of light sensitivity (Shirai et al., 2016; Mandai et al., 2017; Tu et al., 2019).

### Cell Therapy for Neurosensory Deafness

Stem cell therapy also has the potential to treat hearing loss by replacing cochlear hair cells, although this is a difficult environment for this approach. The hostile high potassium luminal fluid (endolymph) environment of the cochlear duct poses significant challenges for cell survival. Further, the robust tight junctions in the auditory epithelium in the organ of Corti make donor cellular integration difficult. A recent article reported the ability of iPSC-derived otic progenitors to form connections with co-cultured spiral ganglion-like cells (Chen et al., 2018). Subsequently, these cells were detached and administered to the inner ear *via* round window injection in hearing loss *Slc26a4* mice. Cells positive for the hair cell marker, *MYO7A* (also causative of Usher 1B), were detected in the organ of Corti, indicating the potential of this method to deliver progenitor cells capable of migrating, differentiating and forming appropriate connections in the inner ear (Chen et al., 2018). However, the progenitors did *not* form an organized stereocilial arrangement, which is critical to hearing, and disrupted in Usher cases (Mathur and Yang, 2015).

### Limitations of Cell Therapies

The development of cellular therapies presents a promising and broad approach to treat a host of sensory diseases regardless of genetic etiology. However, several safety concerns remain to be solved. Of principal concern is the potential for donor cells to cause neoplastic changes in the tissue, particularly those that are derived from stem cells. Mouse embryonic stem cells injected into the subretinal space have been shown to induce tumor formation (Arnhold et al., 2004), though human iPSC-derived photoreceptor progenitor delivery to *rd1* mice found no evidence of abnormal cell growth (Barnea-Cramer et al., 2016). Additionally, Chen et al. (2018) injected mouse models with hiPSC-derived otic progenitors and hiPSC controls to examine the tumorigenicity of hiPSC-derived cells. They reported tumor formation in hiPSC-injected tissue, but not in tissue injected with hiPSC-derived progenitors (Chen et al., 2018). Exclusion of undifferentiated stem cells before transplantation could further decrease the risk of neoplastic changes in the host. In a recent study, Gagliardi et al. (2018) demonstrated the potential of this strategy by safely transplanting CD73+ photoreceptor precursors separated from a cell population using magnetic-activated cell sorting into the eyes of P23H-transgenic rats.

Despite the multitude of challenges in delivering stem cell therapeutics safely and effectively, regenerative medicine remains attractive to patients, industry, and commercial clinics. Several stem cell-based clinical trials are listed on clinicaltrials.gov for AMD (NCT01736059, NCT01920867) and other ocular diseases (NCT01920867), including RP (NCT02320812). Recently, the stem cell ophthalmology treatment study (SCOTS) published results from a phase I/IIa study of five Usher syndrome patients with bilaterally-treated eyes (Weiss and Levy, 2019). Autologous bone marrow-derived stem cells were separated from bone marrow aspirate and administered into patients’ eyes *via* a combination of retrobulbar, sub-Tenon, intravitreal, subretinal, and intra-optic injections, as chosen by the patient and depending on the extent of visual loss and relative risks. Increases in visual acuity were noted in 80% of treated eyes with a statistical significance of *p* < 0.01. There was no reported visual loss nor any complications up to a year post-treatment (Weiss and Levy, 2019). However, a continual follow up is needed to confirm the long-term visual acuity effects and determine whether the patients remain free of adverse events.

## Conclusions

Significant advancements have been made in the last several years towards the treatment and prevention of sensory loss in Usher patients. Particularly, gene and cell therapies pose attractive, potentially one-off solutions that would reduce the burden of invasive re-administration of medications to the eye. Though the majority of studies are currently proof-of-principle treatment strategies using animal models of disease (summarized in Table 3), it is highly possible that the results from current ongoing clinical trials may translate into effective new treatments (see Table 4 for a summary of ongoing clinical trials). However, more temporary therapeutics such as ASOs and translational readthrough inhibitors may also offer a significant reduction in disability. Continuing to improve the safety and efficacy of these treatment approaches is of utmost importance to provide commercially available options for Usher syndrome patients.

**Table 4:** Active clinical trials for Usher patients.

Identifier	Status	Intervention	Usher subtypes	Country
NCT02065011	NR	UshStat—lentiviral delivery of MYO7A	Usher 1B	USA, France
NCT01530659	NR	NT-501—ciliary neurotrophic factor intraocular implant	Usher types 2 and 3	USA
NCT03780257	R	QR-421a—antisense oligonucleotide to induce skipping of exon 13	Usher 2A due to mutations in exon 13	USA, Belgium, Canada, France
NCT03011541	R	Autologous bone marrow-derived stem cells	All	USA

*All trials listed are for the treatment or prevention of Usher-associated retinal degeneration. R, currently recruiting; NR, not recruiting. See www.clinicaltrials.gov for further information*.

## Author Contributions

LF wrote the first draft of the manuscript. FC, CM, and LC contributed to manuscript revision, reading, and approval of the submitted version.

## Conflict of Interest

The authors declare that the research was conducted in the absence of any commercial or financial relationships that could be construed as a potential conflict of interest.

## Footnotes

^1^https://tinyurl.com/r93ejqs

^2^https://www.nature.com/articles/d41586-020-00655-8

^3^https://www.editasmedicine.com

^4^https://ir.editasmedicine.com/news-releases/news-release-details/editas-medicine-reports-recent-progress-jp-morgan-healthcare-0

^5^https://www.proqr.com

^6^https://www.globenewswire.com/news-release/2019/03/11/1751124/0/en/ProQR-Doses-First-Patient-in-Phase-1-2-STELLAR-Trial-of-QR-421a-for-Usher-Syndrome-Type-2.html
